# Prevalence of *Schistosoma haematobium* Infection among School-Age Children in Afar Area, Northeastern Ethiopia

**DOI:** 10.1371/journal.pone.0133142

**Published:** 2015-08-07

**Authors:** Abraham Degarege, Zeleke Mekonnen, Bruno Levecke, Mengistu Legesse, Yohannes Negash, Jozef Vercruysse, Berhanu Erko

**Affiliations:** 1 Aklilu Lemma Institute of Pathobiology, Addis Ababa University, Addis Ababa, Ethiopia; 2 Department of Epidemiology, Robert Stempel College of Public Health and Social Work, Florida International University, Miami, Florida, United States of America; 3 Department of Medical Laboratory Sciences and Pathology, College of Public Health and Medical Sciences, Jimma University, Jimma, Ethiopia; 4 Department of Virology, Parasitology and Immunology, Faculty of Veterinary Medicine, Ghent University, Merelbeke, Belgium; Royal Tropical Institute, NETHERLANDS

## Abstract

In this study, the prevalence and intensity of *Schistosoma haematobium* infection was determined among school-age children living in the Middle and Lower Awash Valley, Afar Regional State of Ethiopia. Between February and May 2014, urine samples were collected from 885 school-age children (5–16 years of age) from the Middle (n = 632; 4 villages) and Lower (n = 253; 3 villages) Awash Valley. All samples were processed using urine filtration to detect and quantify *S*. *haematobium* eggs. In addition, a subset of the urine samples was tested for hematuria using a urine dipstick (n = 556). The overall prevalence was 20.8% (95% Confidence Interval (CI) = 18.1%, 23.5%), based on urine filtration but the prevalence considerably varied across villages both in the Middle (from 12.5% to 37.0%) and Lower Awash Valley (from 0 to 5.3%). The overall mean urine egg count (UEC) among the infected children was 4.0 eggs/10 ml of urine (95% CI = 2.43, 5.52). The infection intensity varied from 0.4 eggs/10 ml of urine to 7.7 eggs/10 ml of urine in the Middle Awash Valley, and from 0 to 1.1 eggs/10 ml of urine in Lower Awash Valley. Age and sex were not associated with *S*. *haematobium* infection based on the multivariable logistic regression model. The prevalence of hematuria was 56.3% (95% CI = 52.2%, 60.4%) among a subset of the study participants (556) examined using the urine dipstick. The prevalence of hematuria also varies with villages from 8.3% to 93.2%. In conclusion, the prevalence of *S*. *haematobium* infection in the Middle Awash Valley was high and it varies across villages. Hence, children living in the present study villages of the Middle Awash Valley need to be treated with praziquantel to reduce morbidity and disrupt transmission.

## Background

Urinary schistosomiasis is a common public health problem in the world caused by infection with *Schistosoma haematobium* [1]. Individuals may acquire the disease during contact with water containing cercaria of the parasite [2]. *S*. *haematobium* is responsible for majority of deaths due to schistosomiasis in the world [3]. The disease is particularly prevalent in sub-Saharan Africa where it is estimated to affect 112 million people [3,4]

*S*. *haematobium* infection causes haematuria, dysuria, lesions of the bladder, kidney failure, bladder cancer, [5–9]. Infection also interferes with nutrient uptake and can lead to undernutrition, growth and cognitive development retardation, and pose a serious threat to children’s health, education and productivity [10–13]. The disease is responsible for the death of 150,000 people in sub-Saharan Africa annually due to infection-related bladder problems [3,4].

In Ethiopia, *S*. *haematobium* infection has been known to be prevalent in northeastern part of Ethiopia following the Awash River Valley, in eastern Ethiopia around Wabi Shebele River and in Kurmuk areas bordering Ethiopia and the Sudan [14–17]. Previous studies in the Afar area documented a prevalence of *S*. *haematobium* ranging from 3.1% to 52.0% [17–20]. Nevertheless, since 1995 there has been limited work on the distribution and prevalence of the disease in the area. As the lifestyle of the communities and environmental conditions favouring survival of the parasite and transmission to human host could vary with time, current information about prevalence of the disease in suspected areas is vital to design appropriate control strategies including preventive chemotherapy of schistosomiasis. Therefore, the objective of the current study was to determine the prevalence and intensity of *S*. *haematobium* infection among school-age children living in different villages of the Amibara District (Middle Awash Valley) as well as in Dubti and Asaita Districts from Lower Awash Valley, Afar Regional State, northeastern Ethiopia.

## Methods

### Study area and population

**Fig 1:**
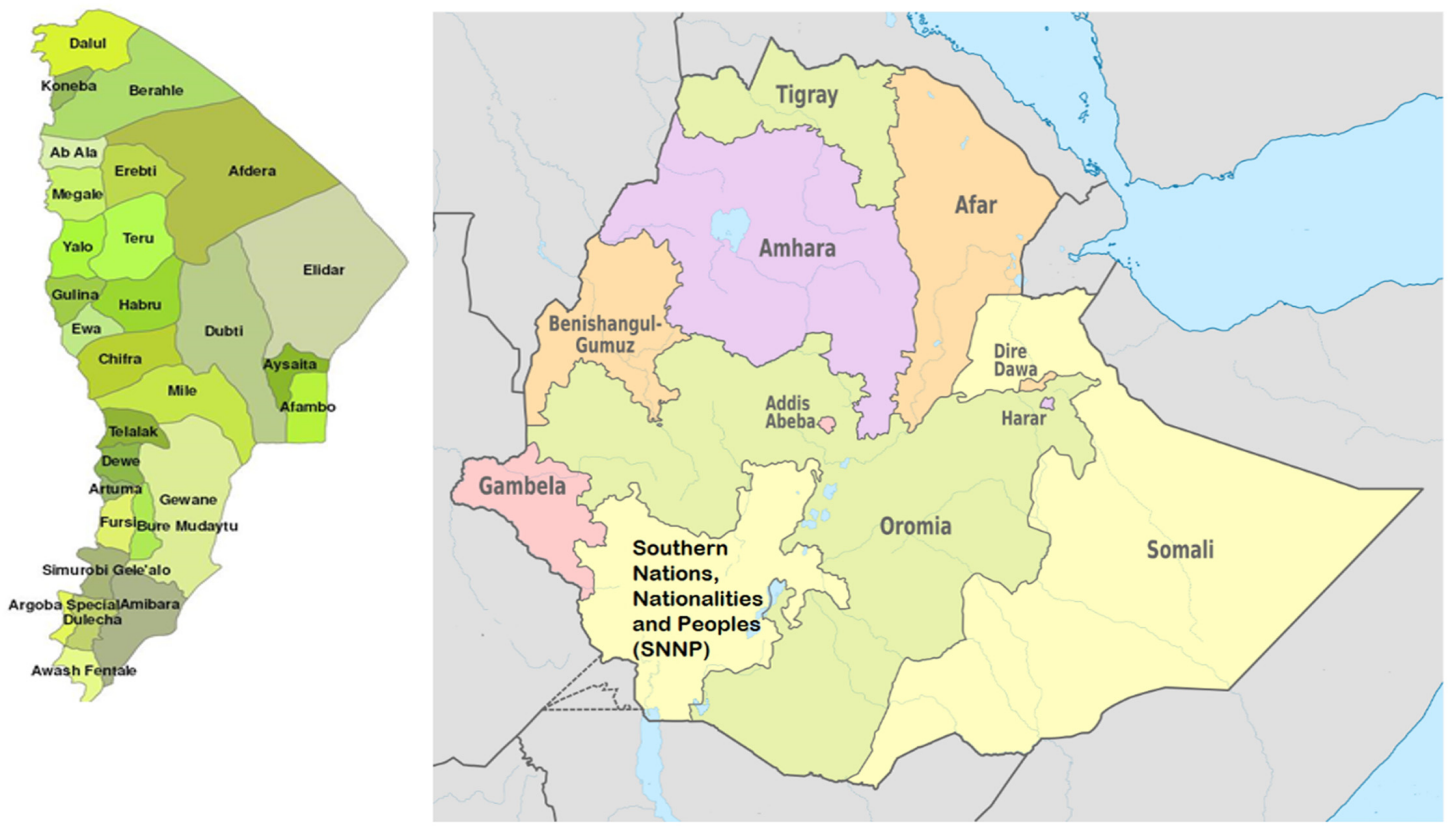
Map of the study region. Map of Afar region Map of Ethiopia.

The study was conducted in 7 purposely-selected villages in the Middle and Lower Awash Valley, Afar Regional State, northeastern Ethiopia (Fig 1). In the Middle Awash Valley, study participants were recruited from four villages of the Amibara District. They are situated about 300 to 350 km from Addis Ababa, and between 720 and 726 m above sea level. The mean annual rainfall and temperature are 654 mm^3^ and 25.6°C, respectively. The majority of the study participants living in the study villages are pastoralists who rely on livestock rearing. A minority are practicing small-scale irrigation to cultivate maize, onion and tomato following the Awash River. There is a large-scale irrigation based cotton farm in the Amibara District of the Middle Awash Valley. The villages in the Lower Awash Valley are located in Asaita District (Berga Debora) and in Dubti District (Farma and Dubti Town). They are situated about 584 (Dubti) to 628 (Asaita) km from Addis Ababa and at altitude between 350 and 381 m above sea level. The mean annual rainfall and temperature are 160 mm^3^ and 32°C, respectively. The pastoralists in the Asaita and Dubti areas practice small-scale irrigation-based cultivation of maize and rear animals. There is a large-scale irrigation-based sugar cane plantation in Dubti area.

The study villages were selected based on their endemicity for *S*. *haematobium* infection [17–20], location near to Awash River, availability of irrigation canals and swamps. Children living in the study villages spend times in the Awash River, irrigation canals and swamps playing, swimming and keeping animals which may expose them to *S*. *haematobium*. Thus, all school age children (5 to 16 years of age) who were living in the selected villages between February and May 2014 were eligible for the study.

### Sample size and study design

The sample size was calculated assuming random sampling and an approximate normality in the distribution of the proportion of events (small population size which was not much larger than the sample size), 95% confidence interval (CI) (Zα/2 = 1.96), 5% margin of error and design effect of 2.5. The design effect was computed as the ratio of true sampling variance to the variance based on assumption of random sampling for proportion 50% of sample size 384. Current status of *S*. *haematobium* infection in Afar region is not known. Thus, the prevalence of *S*. *haematobium* was assumed 50% and the sample size was estimated to be 960. Then, 960 was proportionally divided among the 7 villages based on the size of school age children in each village.

However, the number of school age children living in most of the villages were less than the estimated sample size for the villages. Moreover, due to the nomadic nature of the communities some children did not show up during the study period. Thus, all school age children living in the villages (885) who were willing to participate were included in the study.

After obtaining informed consent from parents/guardians and assent from children, the study participants were given a labelled plastic container (200 ml capacity) and asked to provide a urine sample between 10:00 am and 2:00 pm. About 10 ml of individual’s urine sample was processed by urine filtration method and microscopically examined for *S*. *haematobium* eggs on the spot [21]. Then, *S*. *haematobium* infection intensity was determined as light (1–49 egg/10 ml urine) and heavy (≥50 eggs/10 ml urine) [5]. A subset of the urine samples was also tested for the presence of blood as an indicator of *S*. *haematobium* infection using Combur 10 Test reagent strips (Human GmbH-Max-Planck-Ring 21, Germany) following the manufacturer’s instructions. Briefly, after immersing the test strip in the urine sample for 1 to 2 second, the strips were taken out and left over the bench for about 1 min. Finally, the blood area on the test strip was compared with corresponding reference values (color fields). The results were recorded as 0 (negative), ca.5-10 (weak), ca.50 (moderate) and ca.250 (strong) erythrocytes/μl urine or a haemoglobin concentration out of ca. 10, ca. 50, ca. 250 Ery/μl as per the manufacturer's instruction. The test strip has a minimum sensitivity of 5 to 10 erythrocytes/μl urine corresponding to 0.015 mg haemoglobin or myoglobin/dl urine. Flecked discolorations in the test field indicate intact erythrocytes. A questionnaire was also used to collect information about the education status, places of residence and age of the study participants.

### Statistical data analysis

#### Epidemiology of *S*. *haematobium*

Data were entered into excel-sheet 2007 and analysed using STATA version 11 (Stata Corporation, College Station, Texas, USA). Prevalence and intensity of *S*. *haematobium* infection was calculated for each village, age and sex group. Prevalence of infection was estimated by dividing the number of individuals diagnosed positive by the urine filtration technique with the total number of children examined. The infection intensity was determined by means of urine egg counts (UEC), expressed as number of eggs per 10 ml of urine. Results of individuals who were not infected with *S*. *haematobium* (zero values) were considered when calculating the mean UEC. Multivariable logistic regression analysis was used to estimate the association of age, sex, place of residence and education status with the prevalence of *S*. *haematobium* infection. Multivariable ordinal logistic regression analysis was used to estimate the association between age, sex, place of residence and education status and class intensity of *S*. *haematobium* infection. Zero-inflated negative binomial regression analysis was used to test the difference in the mean UEC between sex or age groups, place of residence and education status.

#### Agreement between diagnostic techniques

The agreement in qualitative and quantitative test results between the urine filtration and dipstick was assessed. The qualitative agreement in test results was determined using Kappa statistic. Kappa values were interpreted as poor (<0.20), fair (0.21–0.40), moderate (0.41–0.60), substantial (0.61–0.80) and perfect (0.81–1.00) [22]. The quantitative agreement was determined using an ordinal logistic regression. In this regression model the outcome variable was the intensity of the colour urine regent strip test, the predictor was the UEC. 95% CI values were estimated for the odds ratio and mean difference values. The level of significance was set at p<0.05.

### Ethical Consideration

The study was conducted after the study protocol was ethically approved by the Institutional Review Board (IRB) of Aklilu Lemma Institute of Pathobiology, Addis Ababa University. Permission to conduct the study was also obtained from the head of the district Health Offices and the principals of the schools and leaders of the villages. Verbal consent for the children was obtained from their parents/guardians. As the study population was mainly illiterate the IRB endorsed oral consenting of the parents or guardians of the children. Seeing that the study involved minimal risk the committee did not require tape recording or any other form of preserving the consenting processes. The children participated in the study on voluntary basis after being informed about the purposes of the study and giving their assent. Children who were positive for *S*. *haematobium* infection were treated with praziquantel (at 40 mg/kg body weight).

## Results

In total, 885 out of 994 eligible individuals provided urine samples. The mean age was 9.8 year (age range: 5–16 years) and majority of the children were males (57.3%). The sample size and the corresponding distribution of age and sex across the different villages are summarized in Table 1. Children living in the Amibara District of the Middle Awash (n = 632) as well as Asaita and Dubti Districts of Lower Awash Valley (n = 253) were examined for *S*. *haematobium* using filtration method.

**Table 1:** Prevalence of *S*. *haematobium* infection among school-aged children in Awash Valley, Afar Regional State, northeastern Ethiopia, February to May 2014.

Variable		Number examined	Percent positive (95% CI)	P-value*
Age in years		885	20.8 (18.1, 23.5)	0.283
Gender	Female	378	21.7 (17.5, 25.9)	
	Male	507	20.1 (16.6, 23.6)	0.391
Villages	Ambash	40	12.5 (1.8, 23.2)	
	Buri	189	24.9 (18.6, 31.1)	
	Hassoba	254	37.0 (31.0, 43.0)	
	Hanledebe	149	23.5 (16.6, 30.4)	<0.001
	Farma	57	5.3 (0.0, 11.2)	
	Dubti	111	0.0 (0.0, 0.0)	
	Asaita	84	0.0 (0.0, 0.0)	
	Non-school attenders	179	29.7 (22.3, 35.7)	
Education	Elementary	628	17.8 (14.8, 20.8)	0.663
	Junior	78	25.6 (15.6, 35.5)	
Valley	Lower Awash	253	1.2 (0.0, 2.5)	<0.001
	Middle Awash	632	28.6 (25.1, 32.2)	

*P-value: adjusted for age, sex, villages and education status: results are generated form one multivariable logistic regression model.

Elementary = grade 1 to 6; Junior: grade 7 to 8.

### Prevalence and intensity of *S*. *haematobium* infection using urine filtration method

The prevalence for the different villages, sex, Valleys are summarized in Table 1. Out of 885 children, 20.8% (95% CI = 18.1, 23.5) were found positive for *S*. *haematobium* infection. The prevalence of *S*. *haematobium* infection was similar when compared between males and females. The odds of *S*. *haematobium* infection were also similar when compared between school attenders and non-school attenders. However, the difference in the prevalence of *S*. *haematobium* infection was significant among the villages and in the Middle and Lower Awash Valleys.

Out of 184 children found positive for *S*. *haematobium* infection, 90.2% had light intensity of infection and 9.8% had heavy intensity of infection (Table 2). The mean UEC among the 885 children examined was 4.0 eggs/10 ml (95% CI = 2.4, 5.5) of urine. There was significant variation in the mean UEC of *S*. *haematobium* among children who lived in different villages, in the Middle and Lower Awash Valley and had different education status. However, the mean UEC and intensity classes of *S*. *haematobium* were comparable among males and females of different age groups.

**Table 2:** Intensity of *S*. *haematobium* infection among school-age children in Middle Awash Valley, Afar Regional State, northeastern Ethiopia, February to May 2014.

Variable		Number examined	Mean UEC (95% CI)	P-value*	Number of light infections	Number of heavy infection	P-value**
Age in Years		885	4.0 (2.4, 5.5)	0.165	166	18	0.593
Gender	Female	378	4.7 (1.7, 7.8)	0.916	73	9	0.766
	Male	507	3.4 (2.0, 4.8)		93	9	
Villages	Ambash	40	0.4 (0.0, 0.7)		4	1	
	Buri	189	4.5 (1.2, 7.9)		43	4	
	Hassoba	254	7.7 (4.4, 11.0)		83	11	
	Hanledebe	149	4.2 (-1.7, 9.9)	<0.001	33	2	0.872
	Farma	57	1.1 (-1.0, 3.1)		2	1	
	Dubti	111	0.0 (0.0, 0.0)		0	0	
	Asaita	84	0.0 (0.0, 0.0)		0	0	
	Non-school	179	8.0 (2.3, 13.6)		44	8	
	attenders						
Education	Elementary	628	2.8 (1.5, 4.1)	0.001	103	9	0.154
	Junior	78	4.4 (-1.6, 10.3)		19	1	
Valley	Lower Awash	253	0.2 (-0.2, 0.7)	0.004	2	164	0.258
	Middle Awash	632	5.5 (3.3, 7.6)		164	17	

P-value*: adjusted for age, sex, villages and education status: results are generated form one Zero-inflated negative binomial regression.

P-value**: adjusted for age, sex, villages and education status: results are generated form one multivariable ordinal logistic regression model.

Elementary = grade 1 to 6; Junior: grade 7 to 8.

### Agreement in diagnostic techniques

A total of 556 samples were examined using both urine dipstick and filtration methods (Table 3). Based on the results of the urine dipstick, 56.3% of the children were positive for hematuria or *S*. *haematobium* infection. About 65.6% of the children positive for infection showed weak colour reaction (ca.5-10), 13.0% showed moderate colour reaction (ca.50) and 16.0% showed strong colour reaction (ca.250). Prevalence of *S*. *haematobium* based on the strip reagent analysis varies significantly among villages (93.2% to 8.3%) and education status (61.9% to 88.5%) (p<0.001). The prevalence of *S*. *haematobium* infection based on the strip reagent analysis was not associated with age and sex. However, the prevalence of infection was higher in females (76.1%) than in males (50.6%) when the analysis was limited to children in the 12 to 16 year age groups. About 24.5% of the 556 children were positive for *S*. *haematobium* when examined using filtration method, and most of the cases (133) were from Middle Awash Valley.

**Table 3:** Prevalence of *S*. *haematobium* infection among 556 school-age children in the Middle and Lower Awash Valley, Afar Regional State, northeastern Ethiopia, February to May 2014.

Urine filtration Method	Urine dipstick analysis		
	Negative	Positive	Total
Negative	228	192	420
Positive	15	121	136
Total	243	313	556

**Fig 2:**
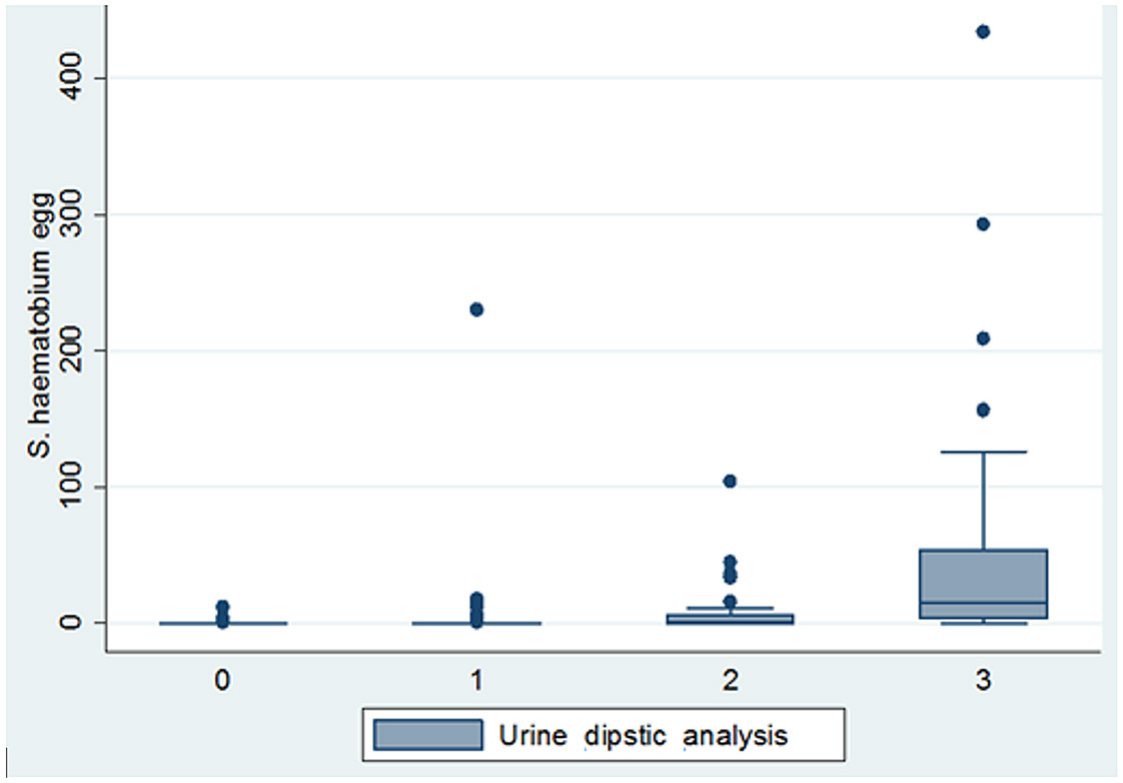
Comparison of the status of *S*. *haematobium* infection based on filtration and dipstick among 556 school-age children in the Middle and Lower Awash Valleys, Afar Regional State, northeastern Ethiopia, from February to May 2014. Urine dipstick analysis: 0 = Negative, 1 = weak (ca.5-10), 2 = moderate (ca.50), 3 = strong (ca.250).

Majority (89.0%) of the children found positive for *S*. *haematobium* based on filtration method were also diagnosed as positive for hematuria by the strip (Fig 2). However, 61.3% of the children detected as positive for hematuria based on the reagent strip had no eggs of *S*. *haematobium* in their urine based on the filtration technique. Majority (88.5%, 170/192) of cases detected as positive for hematuria by the dipstick, but negative for the *S*. *haematobium* by the filtration method had weak colour reaction (ca.5-10). The prevalence of hematuria infection based on dipstick was comparable between males (54.0%) versus females (59.3%) and children of age 5 to 9 (56.5%) and 10 to 16 (56.1%) years. The agreement between the two methods in diagnosing children for the presence of hematuria and *S*. *haematobium* egg was fair [Kappa = 0.3]. The mean UEC estimated with the filtration method was significantly positively associated (predicts) with the intensity grade of colour reaction or hematuria with the reagent analysis [Adjusted Odds Ratio = 1.94, 95% CI = 1.33, 2.55].

## Discussion

The present study was conducted to determine the prevalence of *S*. *haematobium* infection among school-age children living in selected villages in the Middle and Lower Awash Valley, Afar Regional State of Ethiopia. The prevalence of *S*. *haematobium* infection among children in Hassoba, Buri and Hanledebe villages in the Middle Awash Valley was 37%, 25%, and 23.5%, respectively as diagnosed using filtration method. Only three children in Lower Awash Valley (Farma village) were positive for *S*. *haematobium* infection as diagnosed with filtration method. Previous similar study in children living in the two villages of the current study area (Hassoba and Buri) documented a prevalence of 24.5% for *S*. *haematobium* infection [17]. On the other hand, the prevalence of *S*. *haematobium* infection in Hassoba was found to be 47.6% in 2007 [23]. The decline in the prevalence of urogenital schistosomiasis in the study area might be attributed to the drying out of most swamps which support intermediate snail hosts of *S*. *haematobium* (*Bulinus abyssinicus*) and distribution praziquantel drug at the health posts located in the region. Furthermore, the fact that more children are now enrolled at school might have also minimized the chance of exposure of children to cercariae infested swamps as children spend most of their time at school.

Due to difference in behaviour that affects the rate of contact with cercariae infested water, the magnitude of prevalence and intensity of *S*. *haematobium* infection is expected to vary with age and sex. Studies documented an increased prevalence of *S*. *haematobium* infection and mean egg count with an increase in the age of children and in males than in females [24–28]. However, in the current study there was no sex and age-related differences in the prevalence and intensity of infection among children of Middle Awash Valley unlike reports of previous studies [29–31]. Due to differences in environmental setting, culture and religious practices, risk factors of *S*. *haematobium* infection may vary with localities.

There was significant difference in the prevalence and intensity of *S*. *haematobium* infection between children who lived in Middle and Lower Awash Valley and in different villages of the Valley. The intensity of infection was also higher in children who did not attend school compared to those who attended elementary school in the Middle Awash Valley. There are swamps that support intermediate host of *S*. *haematobium* in Hassoba, Hanledebe and Buri. The school-age children may get infected in these swamps. As a result, children living in these villages will continue to be infected and re-infected. Particularly, the rate of infection with the parasite may increase in children who do not attend school as they spend most of their times in swamps when they help their parents with herding outside school activities.

According to WHO guideline of Preventive Chemotherapy in Human Helminthiasis, school-age children living in all of the villages in the Middle Awash Valley (where the prevalence of infection with the parasite lies between 10% and 50%) requires mass drug administration with praziquantel every two years to control schistosomiasis morbidity [32]. However, an integrated approach that may involve provision of clean water, sanitation and hygiene programs; education and behaviour change programs; snail control and development of primary health care systems in the villages would be vital to effectively eliminate urogenital schistosomiasis in the area. Further community based study in these villages would also be vital to determine the status of *S*. *haematobium* infection and risk factors in other age groups to consider them for preventive chemotherapy.

The prevalence of *S*. *haematobium* infection was 24.5% (136/556) in children living in the Middle and Lower Awash Valley based on the filtration method, but increased to 56.3% (313/556) when samples were diagnosed with reagent strip test. Diagnosis of *S*. *haematobium* infection using filtration method may miss cases especially during light infection [33, 34]. Indeed more than 90% of *S*. *haematobium* infection cases in the current study had light intensity of infection and majority (88.5%) of cases detected as positive by the dipstick, but missed by the filtration method had weak colour reaction (ca.5-10). On the other hand, the urine analysis dipstick method will be affected by menstruation and other genitourinary infections [35]. This may contribute to the increased prevalence of infection with the parasite in the region. Indeed in the current study the prevalence of hematuria was significantly higher in females than in males in children with age from 13 to 16 years, but this difference was not maintained in children younger than 13 years. Overall, the results of the current study confirm that the dipstick method could be used for rapid screening of *S*. *haematobium* infection in the region [36]. Ethiopia has launched a National Deworming Programme Targeting School age Children in January 7th, 2015. In this regard, the current finding would be important as it indicates districts and villages in Afar regions where the residents (particularly children) are at risk of schistosomiasis and deworming with praziquantel would be necessary.

The current result is based on urine samples collected only on a single day. On the other hand, the amount of *S*. *haematobium* egg released in urine could vary with days [37]. Had samples collected on different days were examined, the probability of missing light infections could decrease. Thus, the prevalence of *S*. *haematobium* infection in the current study areas might have been underestimated based on the results of the filtration method. Another limitation of the present study is that not all villages suspected for *S*. *haematobium* infection in the Middle and Lower Awash were covered during the survey. Hence, further surveys including all villages in the Middle and Lower Awash Valley are recommended. Female students who had experienced menstruation within recent days before examination were not excluded in the current study. This might have caused overestimation of the prevalence of *S*. *haematobium* infection in females based on the dipstick method.

In conclusion, *S*. *haematobium* infection in the Amibara District of the Middle Awash Valley is fairly high and showed variation in magnitude of prevalence among villages. However, *S*. *haematobium* infection was not common among children in Asaita and Dubti villages of the Lower Awash Valley. According to WHO guideline, school-age children living in endemic villages of the Middle Awash Valley need to be treated with praziquzntel once every two years and other supplementary measures such as provision of clean water and health education are also indicated for eventual elimination of the disease.
